# Supplementary material for: A UMLS-based spell checker for natural language processing in vaccine safety
Source: BMC Med Inform Decis Mak. 2007 Feb 12;7:3. doi: 10.1186/1472-6947-7-3 (PMC1805499; doi:10.1186/1472-6947-7-3)
Supplement: Additional file 13 — RAP application directory. Sets up directory for RAP (RDF application for PHP) source code [file 1472-6947-7-3-S13.gz › rap/doc/phpdoc/model/MemModel.html]

xml version="1.0" encoding="iso-8859-1"?


Docs For Class MemModel


## Class MemModel

Description

Description |
Descendents
| Methods (details)

A MemModel is an RDF Model, which is stored in the main memory.

This class provides methods for manipulating MemModels.

History:

- author: Chris Bizer <chris@bizer.de>
- todo: nothing
- access: public
- author: Gunnar AAstrand Grimnes <ggrimnes@csd.abdn.ac.uk>
- author: Radoslaw Oldakowski <radol@gmx.de>
- author: Tobias Gau� <tobias.gauss@web.de>
- author: Daniel Westphal <mail@d-westphal.de>
- version: V0.9.1

Located in /model/MemModel.php (line 56)

```
Object
   |
   --Model
      |
      --MemModel
```

Direct descendents

Description |
Descendents
| Methods (details)

| Class | Description |
| --- | --- |
| NamedGraphMem | NamedGraph implementation that extends a MemModel with a name. |
| InfModel | A InfModel Model extends a MemModel , by adding the ability to infer statements from known statements and RDFS/OWL-Schematas. |

Method Summary

Description |
Descendents |
Methods (details)

MemModel
MemModel
([string $baseURI = NULL])

void
add
(object Statement $statement)

void
addModel
(object Model &$model)

void
addNamespace
(String $prefix, String $nmsp)

void
addParsedNamespaces
(Array $newNs)

boolean
addWithoutDuplicates
(object Statement $statement)

void
close
()

boolean
contains
(object Statement &$statement)

boolean
containsAll
(object Model &$model)

boolean
containsAny
(object Model &$model)

boolean
equals
(object model &$that)

object MemModel
find
(object Node $subject, object Node $predicate, object Node $object)

object FindIterator
&findAsIterator
([mixed $sub = null], [mixed $pred = null], [mixed $obj = null])

integer
findCount
(object Node $subject, object Node $predicate, object Node $object)

object Statement
findFirstMatchingStatement
(object Node $subject, object Node $predicate, object Node $object, integer $offset)

object MemModel
findRegex
(string $subject\_regex, string $predicate\_regex, string $object\_regex)

object MemModel
findVocabulary
(string $vocabulary)

int
getIndexType
()

Array
getParsedNamespaces
()

object StatementIterator
&getStatementIterator
()

void
index
(int $mode)

object MemModel
&intersect
(object Model &$model)

boolean
isEmpty
()

boolean
isIndexed
()

object FindIterator
&iterFind
([mixed $sub = null], [mixed $pred = null], [mixed $obj = null])

array
rdqlQuery
(string $queryString, [boolean $returnNodes = TRUE])

object RdqlResultIterator
rdqlQueryAsIterator
(string $queryString, [boolean $returnNodes = TRUE])

object MemModel
&reify
()

boolean
remove
(object Statement $statement)

void
removeNamespace
(String $nmsp)

void
replace
(object Node $subject, object Node $predicate, object Node $object, object Node $replacement)

boolean
saveAs
(string $filename, [string $type = 'rdf'])

void
setBaseURI
(string $uri)

integer
size
()

object MemModel
&subtract
(object Model &$model)

string
toString
()

string
toStringIncludingTriples
()

object MemModel
&unite
(object Model &$model)

void
writeAsHtml
()

void
writeAsHtmlTable
()

string
writeRdfToString
()

Methods

Description |
Descendents |
Methods (details)

Constructor MemModel (line 107)

Constructor You can supply a base\_uri

- access: public

MemModel

MemModel
([string $baseURI = NULL])

- string
  $baseURI

add (line 163)

Adds a new triple to the MemModel without checking if the statement is already in the MemModel.

So if you want a duplicate free MemModel use the addWithoutDuplicates() function (which is slower then add())

- throws: PhpError
- access: public

void

add
(object Statement $statement)

- object Statement
  $statement

---

Redefined in descendants as:

- InfModel::add()
  : Adds a new triple to the Model without checking if the statement is already in the Model.
- InfModelB::add()
  : Adds a new triple to the Model without checking, if the statement is already in the Model. So if you want a duplicate free Model use the addWithoutDuplicates() function (which is slower then add())
- InfModelF::add()
  : Adds a new triple to the MemModel without checking if the statement is already in the MemModel.

addModel (line 1139)

Adds another model to this MemModel.

Duplicate statements are not removed. If you don't want duplicates, use unite(). If any statement of the model to be added to this model contains a blankNode with an identifier already existing in this model, a new blankNode is generated.

- throws: phpErrpr
- access: public

void

addModel
(object Model &$model)

- object Model
  $model

---

Redefined in descendants as:

- InfModelF::addModel()
  : Adds another model to this MemModel.

addNamespace (line 1264)

Adds a namespace and prefix to the model.

- access: public
- author: Tobias Gau� <tobias.gauss@web.de>

void

addNamespace
(String $prefix, String $nmsp)

- String
  $prefix
- String
  $nmsp

addParsedNamespaces (line 1250)

Adds the namespaces to the model. This method is called by the parser. !!!! addParsedNamespaces() not overwrites manual added namespaces in the model !!!!

- access: public
- author: Tobias Gau� <tobias.gauss@web.de>

void

addParsedNamespaces
(Array $newNs)

- Array
  $newNs

addWithoutDuplicates (line 202)

Checks if a new statement is already in the MemModel and adds the statement, if it is not in the MemModel.

addWithoutDuplicates() is significantly slower then add(). Retruns TRUE if the statement is added. FALSE otherwise.

- throws: PhpError
- access: public

boolean

addWithoutDuplicates
(object Statement $statement)

- object Statement
  $statement

---

Redefined in descendants as:

- InfModelF::addWithoutDuplicates()
  : Checks if a new statement is already in the MemModel and adds the statement, if it is not in the MemModel.

close (line 1291)

Close the MemModel and free up resources held.

- access: public

void

close
()

contains (line 391)

Tests if the MemModel contains the given triple.

TRUE if the triple belongs to the MemModel; FALSE otherwise.

- access: public

boolean

contains
(object Statement &$statement)

- object Statement
  &$statement

---

Redefined in descendants as:

- InfModelB::contains()
  : Tests if the Model contains the given triple.

containsAll (line 418)

Determine if all of the statements in a model are also contained in this MemModel.

True if all of the statements in $model are also contained in this MemModel and false otherwise.

- access: public

boolean

containsAll
(object Model &$model)

- object Model
  &$model

containsAny (line 443)

Determine if any of the statements in a model are also contained in this MemModel.

True if any of the statements in $model are also contained in this MemModel and false otherwise.

- access: public

boolean

containsAny
(object Model &$model)

- object Model
  &$model

equals (line 979)

Checks if two models are equal.

Two models are equal if and only if the two RDF graphs they represent are isomorphic.

Warning: This method doesn't work correct with models where the same blank node has different identifiers in the two models. We will correct this in a future version.

- throws: phpErrpr
- access: public

boolean

equals
(object model &$that)

- object model
  &$that

find (line 554)

General method to search for triples.

NULL input for any parameter will match anything. Example: $result = $m->find( NULL, NULL, $node ); Finds all triples with $node as object. Returns an empty MemModel if nothing is found.

- throws: PhpError
- access: public

object MemModel

find
(object Node $subject, object Node $predicate, object Node $object)

- object Node
  $subject
- object Node
  $predicate
- object Node
  $object

---

Redefined in descendants as:

- InfModelB::find()
  : General method to search for triples.

findAsIterator (line 1204)

Returns a FindIterator for traversing the MemModel.

- access: public

object FindIterator

&findAsIterator
([mixed $sub = null], [mixed $pred = null], [mixed $obj = null])

---

Redefined in descendants as:

- InfModelB::findAsIterator()
  : Returns a FindIterator for traversing the MemModel.

findCount (line 834)

Searches for triples and returns the number of matches.

NULL input for any parameter will match anything. Example: $result = $m->findCount( NULL, NULL, $node ); Finds all triples with $node as object.

- access: public

integer

findCount
(object Node $subject, object Node $predicate, object Node $object)

- object Node
  $subject
- object Node
  $predicate
- object Node
  $object

findFirstMatchingStatement (line 714)

Searches for triples and returns the first matching statement.

NULL input for any parameter will match anything. Example: $result = $m->findFirstMatchingStatement( NULL, NULL, $node ); Returns the first statement of the MemModel where the object equals $node. Returns an NULL if nothing is found. You can define an offset to search for. Default = 0

- access: public

object Statement

findFirstMatchingStatement
(object Node $subject, object Node $predicate, object Node $object, integer $offset)

- object Node
  $subject
- object Node
  $predicate
- object Node
  $object
- integer
  $offset

---

Redefined in descendants as:

- InfModelB::findFirstMatchingStatement()
  : Searches for triples and returns the first matching statement.

findRegex (line 638)

Method to search for triples using Perl-style regular expressions.

NULL input for any parameter will match anything. Example: $result = $m->find\_regex( NULL, NULL, $regex ); Finds all triples where the label of the object node matches the regular expression. Returns an empty MemModel if nothing is found.

- access: public

object MemModel

findRegex
(string $subject\_regex, string $predicate\_regex, string $object\_regex)

- string
  $subject\_regex
- string
  $predicate\_regex
- string
  $object\_regex

---

Redefined in descendants as:

- InfModel::findRegex()
  : Method to search for triples using Perl-style regular expressions.

findVocabulary (line 670)

Returns all tripels of a certain vocabulary.

$vocabulary is the namespace of the vocabulary inluding a # : / char at the end. e.g. http://www.w3.org/2000/01/rdf-schema# Returns an empty MemModel if nothing is found.

- access: public

object MemModel

findVocabulary
(string $vocabulary)

- string
  $vocabulary

---

Redefined in descendants as:

- InfModel::findVocabulary()
  : Returns all tripels of a certain vocabulary.

getIndexType (line 535)

Returns the indextype: -1 if there is no index, 0 if there are indices over S,P,O(separate),

1. if there is an index over SPO, 2 if there is an index over SP and 3 if

there is an index over SO.

- access: public

int

getIndexType
()

getParsedNamespaces (line 1231)

Returns the models namespaces.

- access: public
- author: Tobias Gau� <tobias.gauss@web.de>

Array

getParsedNamespaces
()

getStatementIterator (line 1192)

Returns a StatementIterator for traversing the MemModel.

- access: public

object StatementIterator

&getStatementIterator
()

---

Redefined in descendants as:

- InfModelB::getStatementIterator()
  : Returns a StatementIterator for traversing the Model.

index (line 479)

Builds a search index for the statements in the MemModel.

The index is used by the find(),contains(),add() and remove() functions. Performance example using a model with 43000 statements on a Linux machine: Find without index takes 1.7 seconds. Indexing takes 1.8 seconds. Find with index takes 0.001 seconds. So if you want to query a model more then once, build a index first. The defaultindex is indices over subject, predicate, object seperate.

mode = 0 : indices over subject,predicate,object (default) mode = 1 : index over subject+predicate+object mode = 2 : index over subject+predicate mode = 3 : index over subject+object

- access: public

void

index
(int $mode)

- int
  $mode

intersect (line 1095)

Returns a new MemModel containing all the statements which are in both this MemModel and another.

- throws: phpErrpr
- access: public

object MemModel

&intersect
(object Model &$model)

- object Model
  $model

isEmpty (line 146)

Checks if MemModel is empty

- access: public

boolean

isEmpty
()

isIndexed (line 517)

Returns true if there is an index, false if not.

- access: public

boolean

isIndexed
()

iterFind (line 1216)

Returns a FindIterator for traversing the MemModel.

- access: public

object FindIterator

&iterFind
([mixed $sub = null], [mixed $pred = null], [mixed $obj = null])

rdqlQuery (line 855)

Perform an RDQL query on this MemModel.

This method returns an associative array of variable bindings. The values of the query variables can either be RAP's objects (instances of Node) if $returnNodes set to TRUE, or their string serialization.

- return: [][?VARNAME] = object Node (if $returnNodes = TRUE) OR array [][?VARNAME] = string
- access: public

array

rdqlQuery
(string $queryString, [boolean $returnNodes = TRUE])

- string
  $queryString
- boolean
  $returnNodes

rdqlQueryAsIterator (line 890)

Perform an RDQL query on this MemModel.

This method returns an RdqlResultIterator of variable bindings. The values of the query variables can either be RAP's objects (instances of Node) if $returnNodes set to TRUE, or their string serialization.

- return: = with values as object Node (if $returnNodes = TRUE) OR object RdqlResultIterator = with values as strings if (if $returnNodes = FALSE)
- access: public

object RdqlResultIterator

rdqlQueryAsIterator
(string $queryString, [boolean $returnNodes = TRUE])

- string
  $queryString
- boolean
  $returnNodes

reify (line 1174)

Reifies the MemModel.

Returns a new MemModel that contains the reifications of all statements of this MemModel.

- access: public

object MemModel

&reify
()

remove (line 227)

Removes the triple from the MemModel.

TRUE if the triple is removed. FALSE otherwise.

- throws: PhpError
- access: public

boolean

remove
(object Statement $statement)

- object Statement
  $statement

---

Redefined in descendants as:

- InfModelB::remove()
  : Removes the triple from the MemModel.
- InfModelF::remove()
  : Removes the triple from the MemModel.

removeNamespace (line 1275)

removes a single namespace from the model

- access: public
- author: Tobias Gau� <tobias.gauss@web.de>

void

removeNamespace
(String $nmsp)

- String
  $nmsp

replace (line 910)

General method to replace nodes of a MemModel.

NULL input for any parameter will match nothing. Example: $m->replace($node, NULL, $node, $replacement); Replaces all $node objects beeing subject or object in any triple of the MemModel with the $needle node.

- throws: PhpError
- access: public

void

replace
(object Node $subject, object Node $predicate, object Node $object, object Node $replacement)

- object Node
  $subject
- object Node
  $predicate
- object Node
  $object
- object Node
  $replacement

---

Redefined in descendants as:

- InfModel::replace()
  : General method to replace nodes of a MemModel.

saveAs (line 357)

Saves the RDF,N3 or N-Triple serialization of the MemModel to a file.

You can decide to which format the model should be serialized by using a corresponding suffix-string as $type parameter. If no $type parameter is placed this method will serialize the model to XML/RDF format. Returns FALSE if the MemModel couldn't be saved to the file.

- access: public

boolean

saveAs
(string $filename, [string $type = 'rdf'])

- string
  $filename
- string
  $type

---

Redefined in descendants as:

- InfModelB::saveAs()
  : Saves the RDF,N3 or N-Triple serialization of the full InfModelB (including inferred triples) to a file.

setBaseURI (line 119)

Set a base URI for the MemModel.

Affects creating of new resources and serialization syntax. If the URI doesn't end with # : or /, then a # is added to the URI.

- access: public

void

setBaseURI
(string $uri)

- string
  $uri

size (line 136)

Number of triples in the MemModel

- access: public

integer

size
()

---

Redefined in descendants as:

- InfModelB::size()
  : Number of all inferable triples in the Model.

subtract (line 1057)

Returns a new MemModel that is the subtraction of another model from this MemModel.

- throws: phpErrpr
- access: public

object MemModel

&subtract
(object Model &$model)

- object Model
  $model

toString (line 281)

Short Dump of the MemModel.

- access: public

string

toString
()

---

Redefinition of:

Object::toString()
:   Serializes a object into a string

---

Redefined in descendants as:

- InfModelB::toString()
  : Short Dump of the InfModelB.
- InfModelF::toString()
  : Short Dump of the InfModelF.

toStringIncludingTriples (line 291)

Dumps of the MemModel including all triples.

- access: public

string

toStringIncludingTriples
()

---

Redefined in descendants as:

- InfModelB::toStringIncludingTriples()
  : Dumps of the InfModelB including ALL inferable triples.

unite (line 1021)

Returns a new MemModel that is the set-union of the MemModel with another model.

Duplicate statements are removed. If you want to allow duplicates, use addModel() which is much faster.

The result of taking the set-union of two or more RDF graphs (i.e. sets of triples) is another graph, which we will call the merge of the graphs. Each of the original graphs is a subgraph of the merged graph. Notice that when forming a merged graph, two occurrences of a given uriref or literal as nodes in two different graphs become a single node in the union graph (since by definition they are the same uriref or literal) but blank nodes are not 'merged' in this way; and arcs are of course never merged. In particular, this means that every blank node in a merged graph can be identified as coming from one particular graph in the original set of graphs.

Notice that one does not, in general, obtain the merge of a set of graphs by concatenating their corresponding N-triples documents and constructing the graph described by the merged document, since if some of the documents use the same node identifiers, the merged document will describe a graph in which some of the blank nodes have been 'accidentally' merged. To merge Ntriples documents it is necessary to check if the same nodeID is used in two or more documents, and to replace it with a distinct nodeID in each of them, before merging the documents. (Not implemented yet !!!!!!!!!!!)

- throws: phpErrpr
- access: public

object MemModel

&unite
(object Model &$model)

- object Model
  $model

writeAsHtml (line 307)

Writes the RDF serialization of the MemModel as HTML.

- access: public

void

writeAsHtml
()

---

Redefined in descendants as:

- InfModelB::writeAsHtml()
  : Writes the RDF serialization of the Model including ALL inferable triples as HTML.

writeAsHtmlTable (line 322)

Writes the RDF serialization of the MemModel as HTML table.

- access: public

void

writeAsHtmlTable
()

---

Redefined in descendants as:

- InfModelB::writeAsHtmlTable()
  : Writes the RDF serialization of the Model including ALL inferable triples as HTML table.

writeRdfToString (line 335)

Writes the RDF serialization of the MemModel as HTML table.

- access: public

string

writeRdfToString
()

---

Redefined in descendants as:

- InfModelB::writeRdfToString()
  : Writes the RDF serialization of the Model including ALL inferable triples.

#### Inherited Methods


Inherited From Model

> Model::Model()  
> Model::findForward()  
> Model::getBaseURI()  
> Model::getMemModelByRDQL()  
> Model::getOntModel()  
> Model::getResmodel()  
> Model::load()  
> Model::visualize()

Inherited From Object

> Object::toString()

Documentation generated on Thu, 7 Jul 2005 13:42:00 +0200 by phpDocumentor 1.3.0RC3
